# Response Surface Methodology for Optimization of Multiplex-PCR Protocols for Detection of TYLCV, TSWV and *Fol* Molecular Markers: Analytical Performance Evaluation

**DOI:** 10.3390/genes14020337

**Published:** 2023-01-28

**Authors:** Richecarde Lafrance, José Benigno Valdez-Torres, Claudia Villicaña, Raymundo Saúl García-Estrada, Mayra Janeth Esparza-Araiza, Josefina León-Félix

**Affiliations:** 1 Centro de Investigación en Alimentación y Desarrollo (CIAD), A.C., Culiacán 80110, Sinaloa, Mexico; 2 CONACYT-Centro de Investigación en Alimentación y Desarrollo, A.C., Culiacán 80110, Sinaloa, Mexico; 3 Centro de Innovación y Transferencia de Tecnología Agropecuaria de Sinaloa- Fundación Produce Sinaloa, A.C., Aguaruto 80308, Sinaloa, Mexico

**Keywords:** tomato, molecular markers, resistance genes, protocols, multiplex PCR

## Abstract

Molecular markers linked to disease resistance genes which affect economically important crops are of great interest. In the case of tomato, a major focus on resistance breeding to multiple fungal and viral pathogens such as *Tomato yellow leaf curl virus* (TYLCV), *Tomato spotted wilt virus* (TSWV) and *Fusarium oxysporum* f. sp. *lycopersici* (*Fol*), have led to the introgression of several resistance genes; therefore, molecular markers have become important in molecular-assisted selection (MAS) of tomato varieties resistant to those pathogens. However, assays that allow simultaneous evaluation of resistant genotypes, such as multiplex PCR, need to be optimized and evaluated to demonstrate their analytical performance, as many factors can affect them. This work aimed to generate multiplex PCR protocols for the joint detection of the molecular markers associated with pathogen resistance genes in tomato plants that are sensitive, specific and repeatable. For the optimization a central composite design of a response surface methodology (RSM-CCD) was used. For analytical performance evaluation, specificity/selectivity and sensibility (limit of detection and dynamic range) were analyzed. Two protocols were optimized: the first one with a desirability of 1.00, contained two markers (At-2 and P7-43) linked to *I-* and *I-3*-resistant genes. The second one with a desirability of 0.99, contained markers (SSR-67, SW5 and P6-25) linked to *I-, Sw-5-*, and *Ty-3*-resistant genes. For protocol 1, all the commercial hybrids (7/7) were resistant to *Fol,* and for protocol 2, two hybrids were resistant to *Fol*, one to TSWV and one to TYLCV with good analytical performance. In both protocols, the varieties considered susceptible to the pathogens, no-amplicon or susceptible amplicons, were observed. The optimized multiplex PCR protocols showed dynamic ranges from 5.97 up to 161.3 ng DNA. The limit of detection was 17.92 ng and 53.76 ng DNA for protocols 1 and 2, respectively, giving 100% positive results in the test replicates. This method allowed to develop optimized multiplex PCR protocols with few assays which translates into less time and resources, without sacrificing method performance.

## 1. Introduction

Tomato is common worldwide and has become an economically important crop due to the benefits of its fruit (a red berry of variable shape and size), rich in carotenoids, flavonoids, and vitamins; hence, it is sold and consumed both fresh and processed [1]. In Mexico, tomato is one of the main vegetables for export and domestic consumption. It is grown in most of the northwestern and western states of the country. In 2020, Mexico produced 3,441,639 tons of tomato, ranking ninth in worldwide production [2]. However, multiple fungal and viral pathogens such as TYLCV, TSWV and *Fol* affected its production, causing diseases that reduce yields, fruit quality and nutritional content. In extreme cases, these diseases have forced growers to abandon production altogether [3,4,5,6]. Treatments of these diseases have mainly been chemical. However, high dependence on pesticides presents health risks to farmers, their families, and consumers. Furthermore, it negatively affects the environment, and substantially increases production costs, which increases financial risks for farmers and leads to higher costs for consumers [7].

To achieve sustainable agriculture and obtain high-quality products in terms of food safety, the use of resistant varieties has been implemented as a high-impact tool to reduce the damage caused by pathogens [8,9]. This can be achieved by genetic improvement through molecular markers, which allow the detection and identification of genes of interest in plants at early developmental stages, taking less time than conventional morphological markers. Moreover, it is not affected by epigenetic changes such as DNA methylation, histone modification and microRNAs [10]. Different types of techniques are used to detect molecular markers and allow the analysis of variation in the DNA molecule; restriction, and different types of Polymerase Chain Reaction (PCR) are some of them, with multiplex PCR being one of the most frequently used to identify molecular markers by using primer sets within a single PCR mix to produce amplicons of different sizes. This variant of PCR targets multiple markers or genes at once in a single test that would otherwise require several times the reagents and more time to run [11,12]. 

The endpoint multiplex PCRs provide a relatively easy, fast, and reliable method for the ensemble detection of molecular markers that serve as “signs” or “flags” of target genes, reducing the workload and costs of marker-assisted selection. The amplicons are visualized by gel electrophoresis in the case of endpoint multiplex PCR, which is different in real-time multiplex PCR where intercalating dyes such as EVA Green and SYBR Green I are used [13,14]. However, the success of these procedures is highly dependent on the concentration of the components and the reaction conditions. This work aimed to generate optimal, validated, and implementable protocols for the joint detection of molecular markers associated with pathogen resistance genes in tomato plants that are specific, sensitive, and reproducible. The development of these protocols may be useful for better selection of resistant materials by growers. In addition, the optimized protocols could be used in future genetic selection procedures of tomato lines for the genetic improvement of the species.

## 2. Materials and Methods

### 2.1. Plant Varieties

Ten tomato varieties were tested, where seven of them were commercial hybrids with declared resistance by the suppliers to pathogens such as TYLCV, TSWV and *Fol* races 1, 2 and 3; and three tomato varieties kindly provided by the Agronomy Faculty of UAS (Table 1). The seeds were grown under greenhouse conditions (daytime temperatures of 21 °C to 27 °C, and night-time temperatures of 16 °C to 18 °C). Three to four weeks after planting, young leaves were sampled by taking at least three replicates of each material, transported on ice, and stored at 80 °C until DNA extraction.

### 2.2. DNA Extraction

DNA was extracted from approximately 200 mg of young leaves ground in liquid nitrogen using a slightly modified 2.5% Cetyl trimethyl ammonium bromide (CTAB) method [15]. The integrity of the Genomic DNA was assessed by running a 0.8% agarose gel in Tris-Acetate-EDTA (TAE), then stained with ethidium bromide and visualized under UV light with an Axygen system (Corning, Axygen^®^ CTR, Mexico). DNA concentration and purity was measured using a QIAxpert system (Qiagen) considering 260/280 nm ratios around 1.8 as accepted DNA, and ratios lower than this value as proteins and phenol contamination.

### 2.3. Molecular Markers Selection Criteria

One simple sequence repeat (SSR) and four sequence characterized amplified region (SCAR) markers were used as candidates for the multiplex PCRs assay, testing two tomato viruses and one fungus resistance locus (Table 2). For the multiplex assay, PCR markers were chosen considering their diagnostic capacity, simple band patterns, clear size differences between the bands (avoiding similar-in-size amplicons for one given PCR protocol), and their capacity to indicate susceptibility and resistance alleles. The multiple primer analyzer program of ThermoFisher Scientific was used for the detection of possible primer dimer formation during the multiplex PCR.

### 2.4. Central Composite Design and Optimization

The protocols were optimized using DNA of three tomato genotypes (SVTE8444 for protocol 1, and a mixture of Vanessa and D-74 as template for protocol 2) carrying different combinations of *Fol*, TYLCV and TSWV resistance gene alleles in homozygous and heterozygous states. For the optimization of the multiplex PCR, the effects of the four independent factors (temperature of annealing (Ta), DNA amount, MgCl_2_, and primer concentrations) were investigated using a response surface methodology (RSM) (Table 3). The central composite design (CCD) for RSM required five levels, coded as −2, −1, 0, 1, 2. The total number of trials was 30, based on the five levels and a four-factor experimental design, with six replicates at the central conditions of the design for estimation of a pure error sum of squares (Supplementary Table S1). The levels for Ta were defined using a central condition for the annealing temperatures reported in previous studies where these markers have already been used, although the DNA amount, MgCl_2_, and primer concentrations of centrals and axial points were calculated based on the recommended PCR kit specifications. Multiplex PCR reactions were performed in a thermal cycler (Bio-Rad) using the GoTaq PCR Core Systems in a final volume of 25 µL reaction containing 1X GoTaq reaction buffer, 0.6 mM dNTPs and 1 U *Taq* polymerase. The dependent variable was the absolute difference of fluorescence intensity between the amplicons measured as pixels (denoted as Adj. Vol. in the software Image Lab (Bio-Rad)), considering that we looked for conditions where the reactions showed the greatest similarity in the intensity of the amplifications which indicates similarity in the opportunity of the primers to align. Considering significant differences at *p* < 0.05, a full quadratic regression model was estimated, and the statistical significance and goodness of fit was evaluated by analysis of variance (ANOVA) and R-squared (R^2^). Optimization was computed using a response optimal analysis (contour plots and the desirability function) and verified using SVTE8444 (protocol 1), D-74, and Vanessa (protocol 2) hybrids’ DNA. All collected data were analyzed using the statistical package MINITAB 18.1.

### 2.5. Multiplex PCR Conditions

For all markers’ amplification, the following conditions were used: denaturation, 4 min at 94 °C; 34 cycles consisting of 30 s at 94 °C, 60 s at Ta shown in Table 3, and 60 s at 72 °C; and final extension of 10 min at 72 °C.

### 2.6. Dynamic Range and Limit of Detection

The limit of detection is the minimum concentration at which a positive sample yields positive results at least 95% of the time. The dynamic range is the interval within which the analyte (amplicons) is detectable [21]. To determine the dynamic range of the markers in both protocols of multiplex PCR, serial dilutions of DNA from the same hybrids used in each optimized protocol were performed in a 1:3 ratio starting from 483.9 ng until 1.99 ng. The limit of detection was determined by testing five replicates with the lowest DNA amount in which the markers amplified, with at least 90% positive replicates. For each reaction, 1 μL of DNA was added in a final volume of 25 μL under the conditions previously described. multiplex PCR products were size-separated on a 1% agarose gel in 1×TAE buffer at 70 V, stained with ethidium bromide and visualized under UV light with the Axygen system (Corning, Axygen^®^ CTR, Culiacan, Sinaloa, Mexico).

### 2.7. Specificity/Selectivity

To evaluate specificity/selectivity, detection of molecular marker sequences was performed using genomic DNA from all commercial and non-commercial tomato varieties (Table 1) using the optimized multiplex PCR protocols.

## 3. Results

### 3.1. Optimization of the Multiplex PCRs Conditions

**Figure 1:**
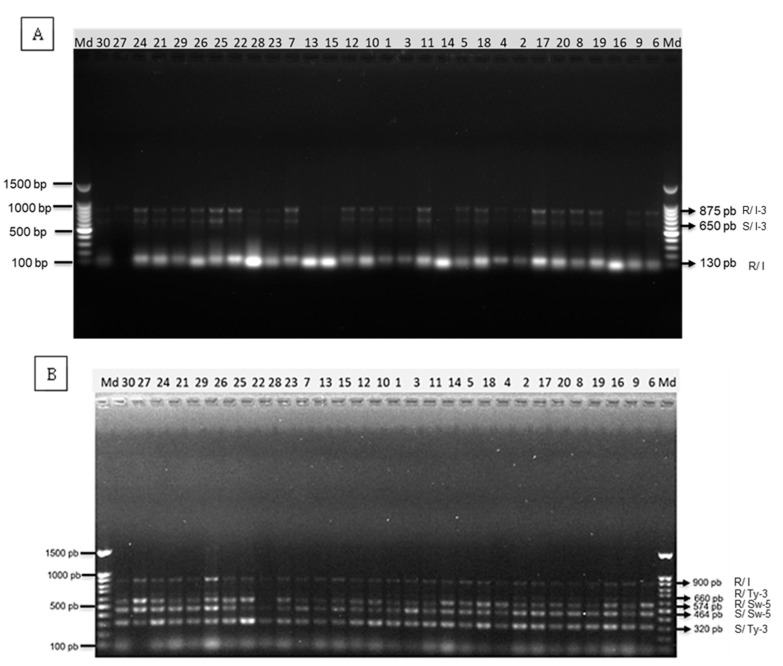
A 1% agarose gel electrophoresis of the amplifications (**A**) At-2 and P7-43; (**B**) SSR-67, Sw-5 and P6-25 markers. Md: 100 bp molecular weight marker. Number 1–30: Standard order of the runs (optimization).

The multiplex PCR runs, with primer pair mixing, was able to identify the specific region associated with resistance expression, without competition or inhibition between primers. For the At-2 marker of the *I* gene and P7-43 marker of the *I-3* gene, resistant alleles (130 bp) and heterozygous-resistant alleles (875/650 bp) were observed in most of the runs using SVTE8444 as a template (Figure 1A). In the case of the SSR-67 marker of the *I* gene, the SW-5 marker of *Sw-5* gene and P6-25 marker of *Ty-3* gene resistant allele (900 bp), heterozygous-resistant alleles (574/464 bp) and heterozygous-resistant alleles (660/320 bp) were observed in most of the runs, respectively, using a mixture of Vanessa and D-74 as a template (Figure 1B).

**Figure 2:**
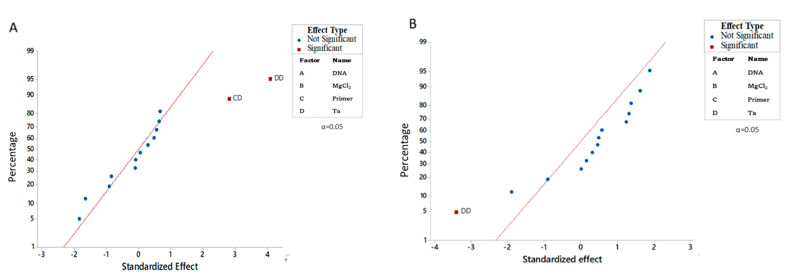
Statistically significant and not significant differences between concentrations of each factor and their interaction, normal plot of the standardized factors and interaction effects on the response variable (fluorescence intensity of the PCR products). (**A**) Multiplex PCR of At–2, and P7–43, molecular markers; (**B**) multiplex PCR of SSR–67, Sw–5 and P6–25 molecular markers.

Statistical analysis of the tested factors showed that the square of annealing temperature and the interaction between the primers’ concentration and annealing temperature were significant, indicating stronger effects over fluorescence intensity for At-2 and P7-43 markers (Figure 2A, Table 4). For markers SSR-67, SW5 and P6-25 the square of annealing temperature was the only significant factor (Figure 2B, Table 4).

**Figure 3:**
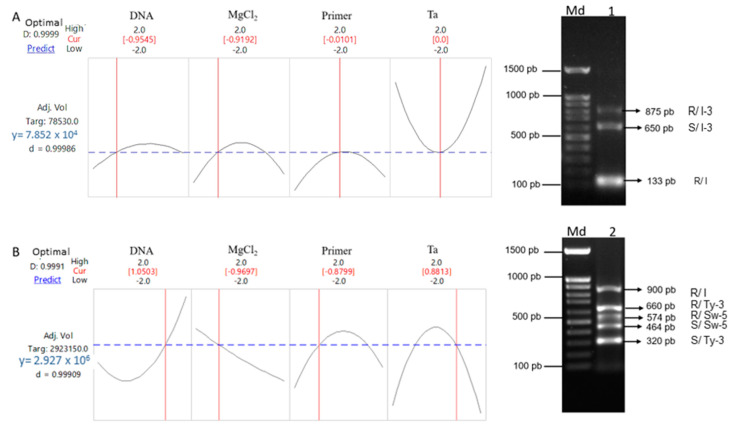
Desirability function plots of optimized PCR for molecular markers (**A**) At–2, and P7–43, (**B**) SSR–67, Sw–5 and P6–25. Md, 100 bp molecular weight marker. R indicates resistant genotype and S indicates susceptible genotype. The 1 indicates protocol 1 and 2 indicates protocol 2.

Since only one of the interactions in the first protocols was significant, near-optimal settings for both protocols were calculated, choosing the trial in which a minimum difference in fluorescence intensity between the amplicons was obtained to favor the amplification of all amplicons. For markers At-2 and P7-43: Ta = 54 °C, 30.5 ng of DNA, 2.02 mM MgCl_2_ and 0.5 µM primers with desirability of 1.00 (Figure 3A). For markers SSR-67, SW5 and P6-25: Ta = 55.88 °C, 71 ng of DNA, 2.00 mM MgCl_2_ and 0.31 µM primers with desirability of 0.99 (Figure 3B). With amplification of all the expected fragments, we did not observe any lack of specificity when validating the optimized multiplex PCRs.

### 3.2. Dynamic Range, Limit of Detection and Specificity/Selectivity

**Figure 4:**
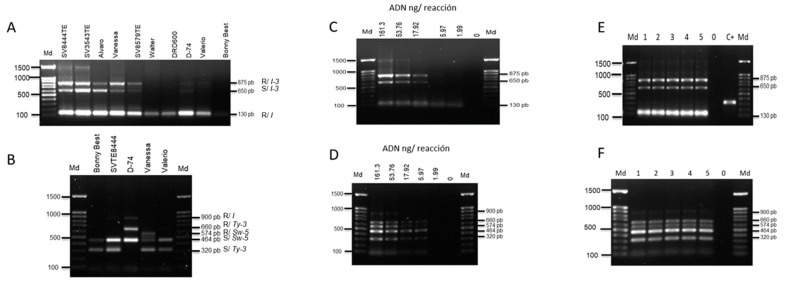
Evaluation of dynamic range and specificity/selectivity of optimized PCR protocols. Specificity/selectivity: (**A**,**B**); dynamic range: (**C**,**D**); limit of detection: (**E**,**F**). Md, 100 bp molecular weight marker. R indicates resistant genotype and S indicates susceptible genotype. C+, positive process control (plasmid DNA).

For protocol 1, in all the commercial hybrids (7/7) considered resistant to *Fol* 1 and *Fol* 3, amplification of resistant alleles was observed. The tomato variety Bonny Best as expected did not present any marker associated with the resistance genes (Figure 4A).

For protocol 2, hybrids SV8444TE and D-74 showed resistance patterns to *Fol* by amplifying the 900 pb representative of the SSR-67 marker which served to discriminate the resistant/tolerant cultivar with an allele size of 900 pb in comparison to the susceptible one with no amplicon. The commercial hybrid Vanessa was the only tomato variety which showed the resistance allele of a SW-5 marker. The hybrid D-74 also showed a resistance allele of 660 bp of a P6-25 molecular marker, and all the other varieties only showed a susceptible allele of 320 bp. The variety Bonny best and the commercial hybrid Valerio amplified the susceptible alleles of SW-5 and P6-25 and no amplicon for SSR-67, which was expected from the Bonny Best but not Valerio (Figure 4B). The optimized PCR protocols showed dynamic ranges from 5.97 ng up to 161.3 ng DNA (Figure 4C,D). The limit of detection was 17.92 ng and 53.76 ng DNA for protocols 1 and 2, respectively, giving 100% positive results in the test replicates (Figure 4E,F).

## 4. Discussion

Without resorting to statistical methods, to optimize a PCR often requires testing a large number of reaction conditions. In the case of multiplex PCR, establishing conditions that allow amplification of multiple molecular markers using primer mixtures requires proportionally more effort and a larger number of reactions. Multiplex PCRs require that primers lead to amplification of single regions of DNA, either in single pairs or in combinations of many primers, under a single set of reaction conditions. Although there is no clear theoretical limit to the number of sequences that can be amplified simultaneously, restrictions in setting conditions for specific and interpretable reactions often limit the useful number of target sequences [22]. Using a response surface methodology reduces the number of reactions, because the program algorithm provides a minimum number of runs considering the most important combinations.

Multiplex PCR can detect a greater total concentration of amplification products than is obtained with a simplex PCR. However, the efficient detection of specific amplicons is greatly influenced by intrinsic parameters related to a Multiplex PCR reaction mixture and amplification conditions, such as the rate at which amplified fragments anneal with each other rather than the primers, reagents concentration, number of cycles, temperature of annealing, among others, which affect Multiplex PCR performance, including reproducibility, sensitivity, and specificity of the technique [22]. In this study, we performed optimization of Multiplex PCR parameters through RSM using a CCD design to model experimental data and found the critical factors that optimized the response variable, in this case, fluorescence intensity of multiplex PCR products. One of the keys to successful multiplex PCRs of any type lies in the design of appropriate primers, even more so in the case of a multiplex PCR. The primer concentration is a critical parameter for a successful multiplex reaction [23]. Ideally, all the primers should enable identical amplification efficiencies for each amplicon. While it is difficult to predict the efficiency that any given primer pair will display, primers with nearly identical optimum annealing temperatures should work under fairly similar conditions if they anneal with single copy sequences. If all the primers in a reaction anneal with equal efficiencies, they can generally be used at the same concentration [21]. Another key parameter for successful multiplex PCRs is the annealing temperature. The optimal Ta directly depends on the composition (GC content) and length of primers and is usually used 5 °C below the melting temperature of primers. Therefore, interactions between the primer and Ta usually affect multiplex PCRs [24].

The primers of the At-2 marker (protocol 1) were designed to amplify a 130 bp band only in resistance strains, since the reverse primer matches with an additional 7 bp present only in resistant varieties and the forward primer matches with a common region in resistant and susceptible varieties [16]. PCR products of 875 bp for resistant and 650 bp for susceptible varieties were reported by Barillas et al. [17] for the P7-43 marker (protocol 1), when they tested the commercial hybrids, Plum Crimson, Amelia, Crista, Solar Fire and three experimental hybrids resistant to *Fol* race 3 (I-3/i-3). In our study, all the commercial hybrids considered resistant to *Fol* analyzed with these molecular markers showed the same resistant alleles. These results demonstrate the good performance of the optimized protocol; in this case, its specificity. Cultivars containing, I-3 have been successfully used for the control of *Fol* 3 for over more than 30 years, but this resistance has not been as ubiquitously deployed as the I or I-2 genes, and therefore, selection pressure for mutation in the pathogen has not been as intense [25]. The validation of a multiplex PCR protocol containing a molecular marker linked to this resistance gene will lead to the increased use of this gene.

SSR-67 (protocol 2) was found to be a putative marker for resistant tomato cultivars and could discriminate between tolerant/resistant cultivars with an allele of 900 bp and no amplicon in susceptible cultivars [18]. For the SW-5 marker (protocol 2), homozygous-resistant, susceptible, and heterozygous-resistant tomato genotypes presented the same band size when analyzed by Kabaş et al. [26] using 40 tomato hybrids. Allele variants of this SCAR codominant marker (SW5) associated with resistance or susceptibility to TSWV are known to have specific sizes [26,27]. The P6-25 marker (protocol 2) based on locus *Ty-3* of the tomato genome is known to be associated with tomato resistance to TYLCV. Tomato hybrids resistant to TYLCV from different commercial seed companies analyzed with the P6-25 marker presented two alleles (660/320 bp) for heterozygous-resistant genotypes [20,28,29,30]. This marker has been widely used for selecting resistant tomato lines [31,32], suggesting that the target region is highly conserved and linked to *Ty-3* mediated resistance. The resistant alleles shown in the considered resistant materials and susceptible alleles or no amplification when it is the case by these molecular markers in our study also demonstrate the specificity of this optimized protocol.

Pyramiding multiple resistance genes against pathogens such as *Fol*, TYLCV and TSW is difficult to achieve by classical breeding. Breeders using MAS can combine multiple resistance genes against them which is likely to extend resistance against multiple fungal and viral strains. The lack of markers in some of the commercial hybrids could probably have been due to the existence of polymorphisms on the target region that likely affected recognition sites of primers [33], suggesting that specificity/selectivity of molecular markers is highly dependent on genetic diversity of the analyzed varieties. Dominant molecular markers such as At-2 and SSR-67 make it difficult to evaluate genotypes as susceptible homozygous and the respective heterozygous in plants. We recommend the use of codominant SCAR molecular markers and tomato materials that allow to evaluate the resistant homozygous, susceptible homozygous and heterozygous, which was one of our limitations during this investigation.

## 5. Conclusions

The optimization of these two protocols using RSM CCD represents an advantageous method for join detection of these molecular markers with good analytical performance using few combinations of the variables that influence multiplex PCR. The present study detected five commonly used molecular makers of tomato linked to resistant genes against economically important diseases in the time and with the reagents (except primers) of two tests, thereby reducing reaction costs and workload. The optimized and validated protocols may be used to identify resistant genotypes for future breeding programs.

## Figures and Tables

**Table 1:** Commercial and non-commercial tomato materials used in this study.

Companies	Hybrids	Declared Resistance by Suppliers	Purposes
Hatear	Álvaro	HR: *Fol* (1,2); Vd; ToMV IR: TSWV; TYLCV; Mi; Mj; ToTV	Validation (protocol 1)
Hazera	Vanessa	HR: *Fol* (1,2,3) TSWV; Pst; ToANV IR: TYLCV	Optimization (protocol 2) and validation (protocols 1 and 2)
Seminis	SV8579TE	HR: *Fol* (0,2); Va; Vd; ToMV IR TYLCV; Mi	Validation (protocol 1)
Seminis	SV3543TE	R: *Fol* (1,2,3); Tmv; TYLCV: ToTV; TYTV	Validation (protocol 1)
Seminis	SVTE8444	IR: *Fol* (1,2,3); TYLCV; Ma; Mi; Mj; Vd	Optimization (protocol 1) and validation (protocols 1 and 2)
Sakata	Valerio	IR: *Fol* (1,2,3); TYLCV; ToMV; Vd R: Aal	Validation (protocol 1 and 2)
No information	DRD600	*Fol* (1)	Validation (protocol 1)
No information	D-74	HR: *Fol* (1,3); TYLCV	Optimization (protocol 2) and validation (protocols 1 and 2)
Victory Seeds	Bonny Best	SC: *Fol* (1, 3); TYLCV	Validation (protocols 1 and 2)
Eden Seeds	Walter	IR: *Fol* (1) (IR)	Validation (protocols 1)

R: resistance; IR: intermediate resistance; HR: high resistance; SC: susceptible; *Fol*: *Fusarium oxysporum* f. sp. *lycopersici*; ToMV: *Tomato mosaic virus*; TSWV: *Tomato Spotted wild virus*; TYLCV: *Tomato yellow leaf curl virus*; ToANV: *Tomato apex necrosis virus*; Pst: *Pseudomonas syringae* pv. *Tomato*; Ma; *Meloidogyne arenaria*; Mi: *Meloidogyne incognita*; Mj: *Meloidogyne incognita*; ToTV: *Tomato torrado virus*; Tmv: *Tobacco mosaic virus*; Aal: *Alternaria alternata* f. sp. *lycopersici*; TYTV: *Tomato yellow top virus*; Va: Vesicular-arbuscular; Vd: *Verticillium dahliae*.

**Table 2:** Gene locus, primers, molecular marker type, annealing temperature, amplicon base pair and references for the *F. oxysporum* f. sp. *lycopersici* molecular markers associated with the resistance genes used in the present study.

Genes	Markers	Types	Chr.	Primer sequences		Ta. (°C)	Amplicons (bp)	References
				Forward	Reverse			
*I*	At-2	SCAR	11	cgaatctgtatattacatccgtcgt	ggtgaataccgatcatagtcgag	54	130 R	Arens et al. [16]
*I-3*	P7-43	SCAR	7	cacgggatatgttrttgataagcatg	gtctttaccacaggaactttatcacc	53	875 R/650 S	Barillas et al. [17]
*I*	SSR-67	SSR	11	gcacgagaccaagcagatta	gggcctttcctccagtagac	--	900 R	Parmar et al. [18]
*Sw5*	SW-5	SCAR	9	aattaggttcttgaagcccatct	ttccgcatcagccaatagtgt	54	575 R/464 S	Dianese et al. [19]
*Ty-3*	P6-25	SCAR	6	ggtagtggaaatgatgctgctc	gctctgcctattgtcccatatataacc	53	660 R/320 S	Ji et al. [20]

Chr.: chromosome; Ta.: annealing temperature; R: resistant; S: susceptible; Bp: base pair.

**Table 3:** Coded variables resulting from the experimental design for endpoint Multiplex-PCR optimization.

Variables	Axial (−2)	Factorial (−1)	Central (0)	Factorial (1)	Axial (2)
Ta. (°C)	50	52	54	56	58
DNA (ng)	10	30	50	70	90
MgCl2 (mM)	1.5	2	2.5	3	3.5
Primer (µM)	0.1	0.3	0.5	0.7	0.9

Ta.: annealing temperature.

**Table 4:** Analysis of variance (ANOVA) for the fluorescence intensity values for molecular markers using RSM-CCD.

Source	DF	AT-2; P7-43				SSR-67; SW5; P6-25			
		Seq SS	% Cont.	*F*	*p*	Seq SS	% Cont.	*F*	*p*
Model	15	53557084617	73.21	2.55	0.044 *	6.297 × 10^13^	73.62	2.60	0.041 *
Blocks	1	857170949	1.17	0.61	0.447	1.463 × 10^13^	17.10	9.07	0.009 *
Linear	4	1463078506	2.00	0.26	0.898	7.391 × 10^12^	8.64	1.15	0.375
A	1	612566451	0.84	0.44	0.519	4.198 × 10^12^	4.91	2.60	0.129
B	1	111438211	0.15	0.08	0.782	22124160	0.00	0.00	0.997
C	1	428883822	0.59	0.31	0.589	3.785 × 10^11^	0.44	0.23	0.635
D	1	310190021	0.42	0.22	0.645	2.815 × 10^12^	3.29	1.75	0.208
Square	4	38452271841	52.56	6.87	0.003 *	3.314 × 10^13^	38.74	5.14	0.009 *
A×A	1	1242705553	1.70	0.73	0.406	1.013 × 10^13^	11.84	3.51	0.082
B×B	1	6742079470	9.22	3.43	0.085	1.109 × 10^12^	1.30	0.02	0.881
C×C	1	7173212681	9.81	2.75	0.120	3.261 × 10^12^	3.81	3.59	0.079
D×D	1	23294274137	31.84	16.64	0.001 *	1.864 × 10^13^	21.79	11.56	0.004 *
2-way int.	6	12784563320	17.48	1.52	0.242	7.816 × 10^12^	9.14	0.81	0.580
A×B	1	11760784	0.02	0.01	0.928	1.331 × 10^12^	1.56	0.83	0.379
A×C	1	18012102	0.02	0.01	0.911	3.289 × 10^11^	0.38	0.20	0.658
A×D	1	1186384543	1.62	0.85	0.373	1.528 × 10^11^	0.18	0.09	0.763
B×C	1	2876642	0.00	0.00	0.964	3.006 × 10^12^	3.51	1.86	0.194
B×D	1	553783263	0.76	0.40	0.539	5.399 × 10^11^	0.63	0.33	0.572
C×D	1	11011745986	15.05	7.87	0.014 *	2.458 × 10^12^	2.87	1.52	0.237
Error	14	19595818329	26.79			2.257 × 10^13^	26.38		
Lack of fit	10	11800089736	16.13	0.61	0.763	1.771 × 10^13^	20.70	1.46	0.382
Pure error	4	7795728593	10.66			4.858 × 10^12^	5.68		
Total	29	73152902946	100.00			8.554 × 10^13^	100.00		

DF, degree of freedom; Seq SS, sequential sums of squares; % Cont., % of contribution; F, F-value; A, DNA; B, MgCl_2_; C, primers; D, temperature of annealing; 2-way int., 2-way interactions. *, indicates significant differences.

## Data Availability

Not applicable.

## Supplementary Materials

The following supporting information can be downloaded at: https://www.mdpi.com/article/10.3390/genes14020337/s1, Table S1: Central composite design runs for the multiplex PCR.
