# Supplementary material for: Response Surface Methodology for Optimization of Multiplex-PCR Protocols for Detection of TYLCV, TSWV and Fol Molecular Markers: Analytical Performance Evaluation
Source: Genes (Basel). 2023 Jan 28;14(2):337. doi: 10.3390/genes14020337 (PMC9956084; doi:10.3390/genes14020337)
Supplement: Supplementary file 1 [file genes-14-00337-s001.zip › genes-2025203-supplementary.pdf]

Table S1. Central composite design runs for the multiplex PCR

StdOrder	Blocks	C. DNA	C. MgCl2	C. Primer	Ta.
30	2	0	0	0	0
27	2	0	0	0	-2
24	2	0	2	0	0
21	2	-2	0	0	0
29	2	0	0	0	0
26	2	0	0	2	0
25	2	0	0	-2	0
22	2	2	0	0	0
28	2	0	0	0	2
23	2	0	-2	0	0
7	1	-1	1	1	-1
13	1	-1	-1	1	1
15	1	-1	1	1	1
12	1	1	1	-1	1
10	1	1	-1	-1	1
1	1	-1	-1	-1	-1
3	1	-1	1	-1	-1
11	1	-1	1	-1	1
14	1	1	-1	1	1
5	1	-1	-1	1	-1
18	1	0	0	0	0
4	1	1	1	-1	-1
2	1	1	-1	-1	-1
17	1	0	0	0	0
20	1	0	0	0	0
8	1	1	1	1	-1
19	1	0	0	0	0
16	1	1	1	1	1
9	1	-1	-1	-1	1
6	1	1	-1	1	-1

Std. Order: Standard order; C. DNA: DNA concentration; C. MgCl2: MgCl2 concentration; C. Primer: Primer concentration; Ta.: annealing temperature.
